# Synthesis and crystal structures of 4,4′-methyl­enebis(2,6-di­ethyl­aniline) and 4,4′-methyl­enebis(3-chloro-2,6-di­ethyl­aniline)

**DOI:** 10.1107/S2056989025000234

**Published:** 2025-01-17

**Authors:** Daniil E. Smirnov, Oleg S. Morozov, Ekaterina S. Afanasyeva, Viktor V. Avdeev

**Affiliations:** a Department of Chemical Technology and New Materials, Faculty of Chemistry, Lomonosov Moscow State University, GSP-1, 1-3, Leninskiye Gory, Moscow, 119991, Russian Federation; Universidad de la Repüblica, Uruguay

**Keywords:** crystal structure, methyl­enedianiline, hydrogen bonding, synthesis, disorder

## Abstract

The title compounds 4,4′-methyl­enebis(2,6-di­ethyl­aniline) (C_21_H_30_N_2_, **1**) and 4,4′-methyl­enebis(3-chloro-2,6-di­ethyl­aniline) (C_21_H_28_Cl_2_N_2_, **2**) are of significant inter­est as curing agents for a wide range of resins and as building blocks for sterically demanding compounds in the synthesis of ligands for catalysis. This paper describes their synthesis and the preparation of single crystals, with their structures determined through single-crystal X-ray analysis. The presence of the chlorine substituent slightly affects the twist angle between the two aromatic components. The mol­ecules of compound **1** form a network structure through inter­molecular N—H⋯N bonds and C—H⋯π inter­actions, while in the crystal structure of compound **2**, the mol­ecules are assembled solely through N—H⋯π inter­actions. Consequently, despite their chemical similarity, it is the precise structural data that enables us to explain their differing reactivity and opens up the possibility of evaluating steric properties for the development of new materials and ligands.

## Abstract 2

Ep­oxy curers 4,4′-methyl­enebis(2,6-di­ethyl­aniline) and 4,4′-methyl­enebis(3-chloro-2,6-di­ethyl­aniline) were prepared and studied by ^1^H NMR and single-crystal X-ray analysis.

## 1. Chemical context

Aromatic di­amines are widely utilized as hardeners for polyurethanes (Ueda *et al.*, 2017 ▸), ep­oxy resins (Yu *et al.*, 2020 ▸; Costa *et al.*, 2005 ▸), cyanate ester (Bauer & Bauer, 2001 ▸) and phthalo­nitriles (Bulgakov *et al.*, 2021 ▸). The reactivity of amines is primarily determined by their nucleophilicity, which depends on the electronic structure and geometry of the mol­ecules. In the production of castings and composite materials, the gelation time of the binder is a critical factor: the longer the gelation time at low temperatures, the more time is available for the impregnation of the reinforcing filler or the moulding of products. Hardeners with low activity at room temperature also facilitate the manufacture of prepregs with a long shelf life. To decrease the reaction rate, the nucleophilicity of the di­amine can be diminished by introducing electron-withdrawing substituents. An example of such a modification is 4,4′-di­amino­diphenyl­sulfone (DDS), which exhibits significantly lower activity than 4,4′-methyl­enedianiline (MDA) (Kong & Park, 2003 ▸). Another strategy for reducing the activity of amines involves the introduction of sterically bulky groups in the *ortho* position, as seen in 4,4′-methyl­enebis(2,6-di­ethyl­aniline) (MDEA). An even more pronounced effect can be achieved by additionally incorporating chlorine into the 3-position of the aromatic ring, specifically in 4,4′-meth­yl­enebis(3-chloro-2,6-di­ethyl­aniline) (MCDEA). In terms of reactivity with ep­oxy monomers, these compounds follow the order: MDEA > DDS > MCDEA (Lahlali *et al.*, 2005 ▸, 2006 ▸). Similarly, in reactions with iso­cyanates, the order of reactivity is MDA > MDEA > MCDEA (Voelker *et al.*, 1988 ▸). Notably, a mol­ecule carrying a methyl group in the *meta* position instead of chlorine, exhibits approximately the same activity as MDEA. Consequently, this raises the question of which factor — steric or electronic – primarily influences the activity of hardeners. Therefore, studying the mol­ecular structure is particularly intriguing for comparing the steric hindrance of the amine in MDEA and MCDEA.
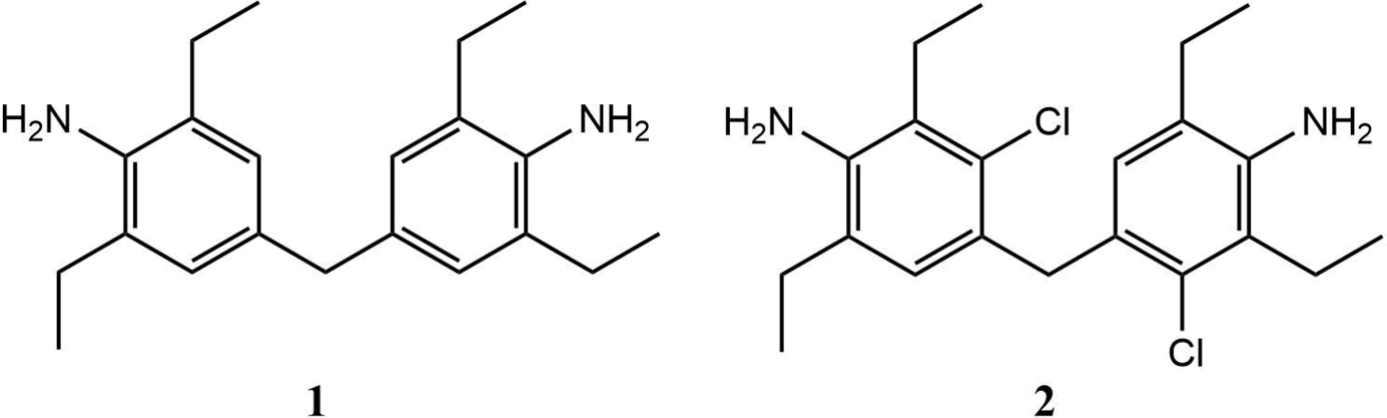


Another potential application of *ortho*-substituted anilines is the synthesis of N-heterocyclic carbenes, including polymeric structures (Peng *et al.*, 2018 ▸). The properties of carbenes as ligands are typically assessed based on the electronic and steric characteristics of the substituents on the nitro­gen atoms (Cavallo *et al.*, 2005 ▸). The steric hindrance is qu­anti­tatively described by the parameter Vbur%, which represents the volume of a sphere occupied by the ligand (Gomez-Suarez *et al.*, 2017 ▸). Mol­ecular structure data obtained from X-ray crystallography can be utilized to calculate Vbur% and more accurately predict the catalytic properties of mol­ecules through machine learning (Escayola *et al.*, 2024 ▸). Consequently, the parameters outlined in this paper will be valuable for various scientists seeking to understand the reactivity of curing agents and predict the properties of structures based on the compounds discussed.

## 2. Structural commentary

**Figure 1:**
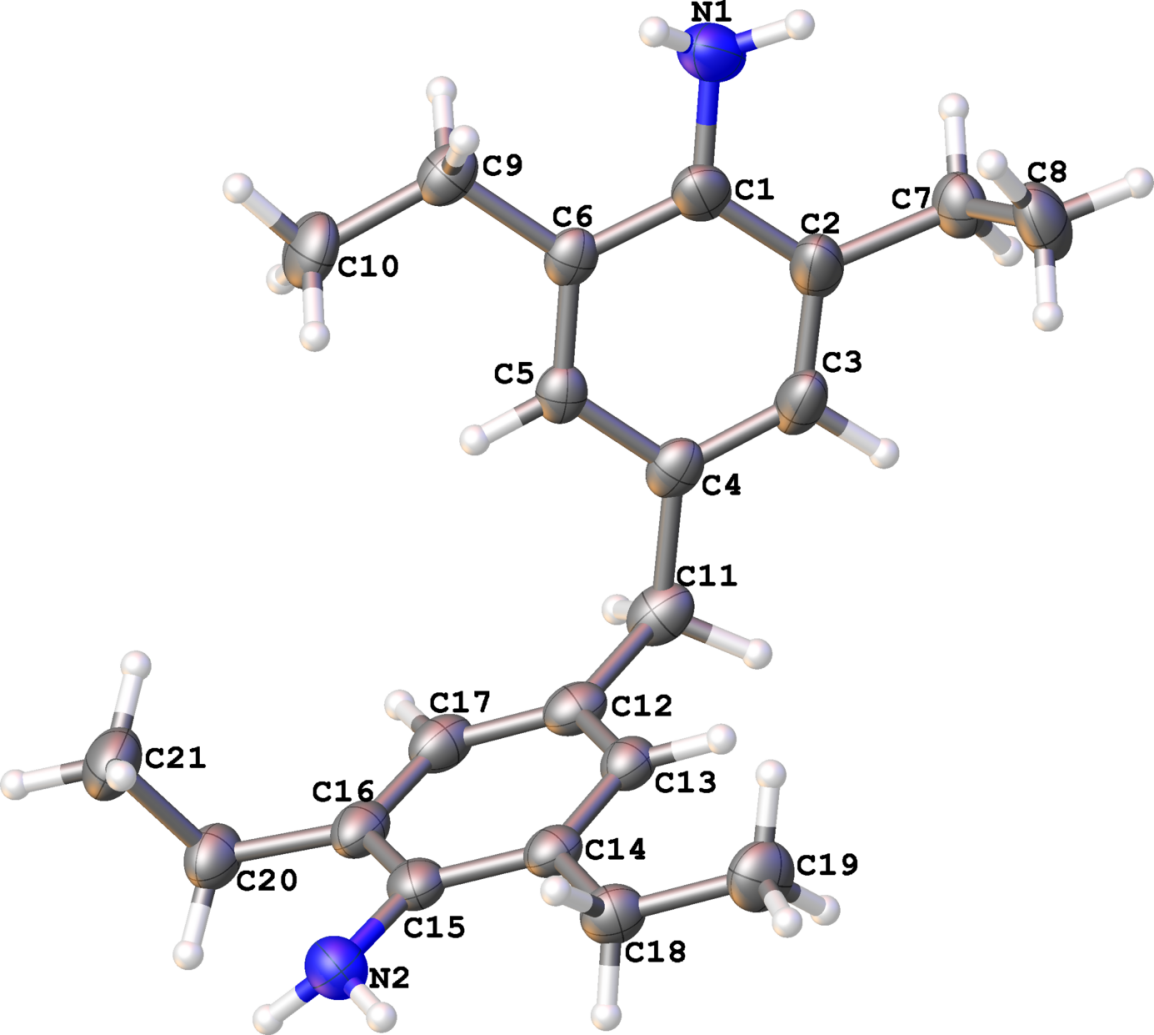
The mol­ecular structure of **1**, with displacement ellipsoids drawn at the 50% probability level. The minor occupancy components are omitted for clarity.

The mol­ecular structures of the title compounds **1** and **2**, which differ in the presence of an additional chlorine substituent at *meta* position to amine, are illustrated in Figs. 1 ▸ and 2 ▸, respectively. In both cases, the organic mol­ecules occupy general positions. Depending on the presence of chlorine substituents, the ring systems are twisted to a greater or lesser extent. In mol­ecule **1**, the dihedral angle between the two aromatic parts is as large as 64.13 (6)°, while the corresponding angle in mol­ecule **2** is 39.59 (8)°. The dihedral angle between those parts approximately equal for both structures [80.24 (4) and 77.72 (6)° for the **1** and **2**, respectively]. In mol­ecule **2**, the chlorine atom in the C12–C17 ring is disordered with an occupancy ratio of 0.920 (2):0.08 (2).

In the unsubstituted mol­ecule **1** the ethyl groups (C9–C10 and C18–C19) are nearly co-planar with their phenyl rings and the ethyl group connected with the C2 atom is disordered. The major components (73.6%) for C7–C8 and ethyl group C20–C21 are directed almost orthogonal [88.6 (4)° and 80.9 (2)°, respectively] to the plane of the phenyl rings, while the minor components are slightly inclined with torsion angle C1—C2—C7′—C8′ = 153.5 (12)°.

The steric hindrance exerted by the chlorine atom in mol­ecule **2** causes the ethyl groups to rotate out of the plane of the phenyl ring. The ethyl groups connected with the C12–C17 phenyl ring are parallel to each other with the torsion angle C19—C18—C20—C21 being 0.60 (17)° while the torsion angle C8—C7—C9—C10 between other ethyl fragments is 116.7 (3)°.

## 3. Supra­molecular features

**Figure 3:**
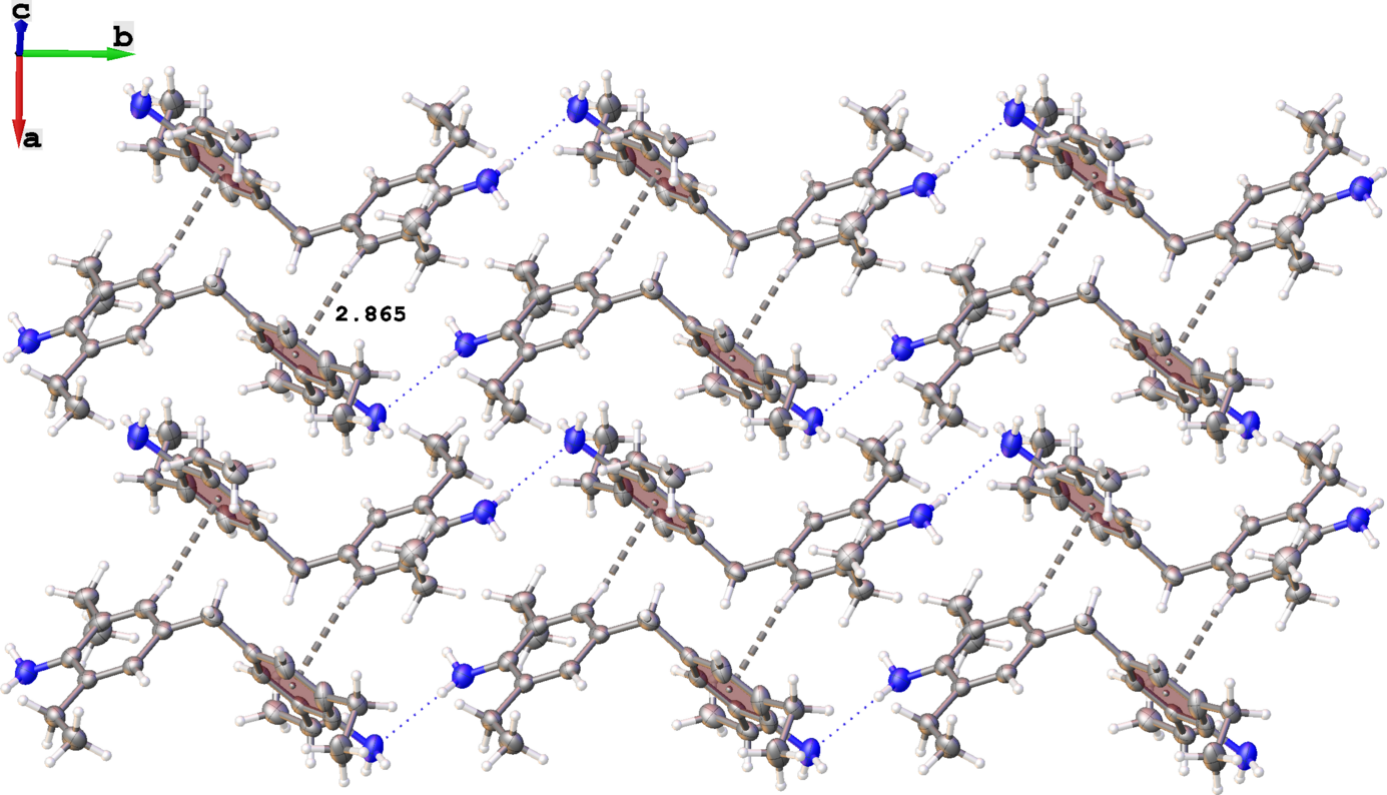
Fragment of the crystal packing of **1**.

In the crystal structure of **1**, the mol­ecules form infinite chains extending along [010] *via* N2—H2*A*⋯N1 hydrogen bonds [N2⋯N1 = 3.472 (2) Å; Fig. 3 ▸, Table 1 ▸]. Additionally, the mol­ecules are grafted together in a herringbone-like manner by C—H⋯π inter­actions [3.8568 (17) Å] involving the phenyl H17 atom and the centroids of the C1–C6 phenyl rings of adjacent mol­ecules.

**Figure 4:**
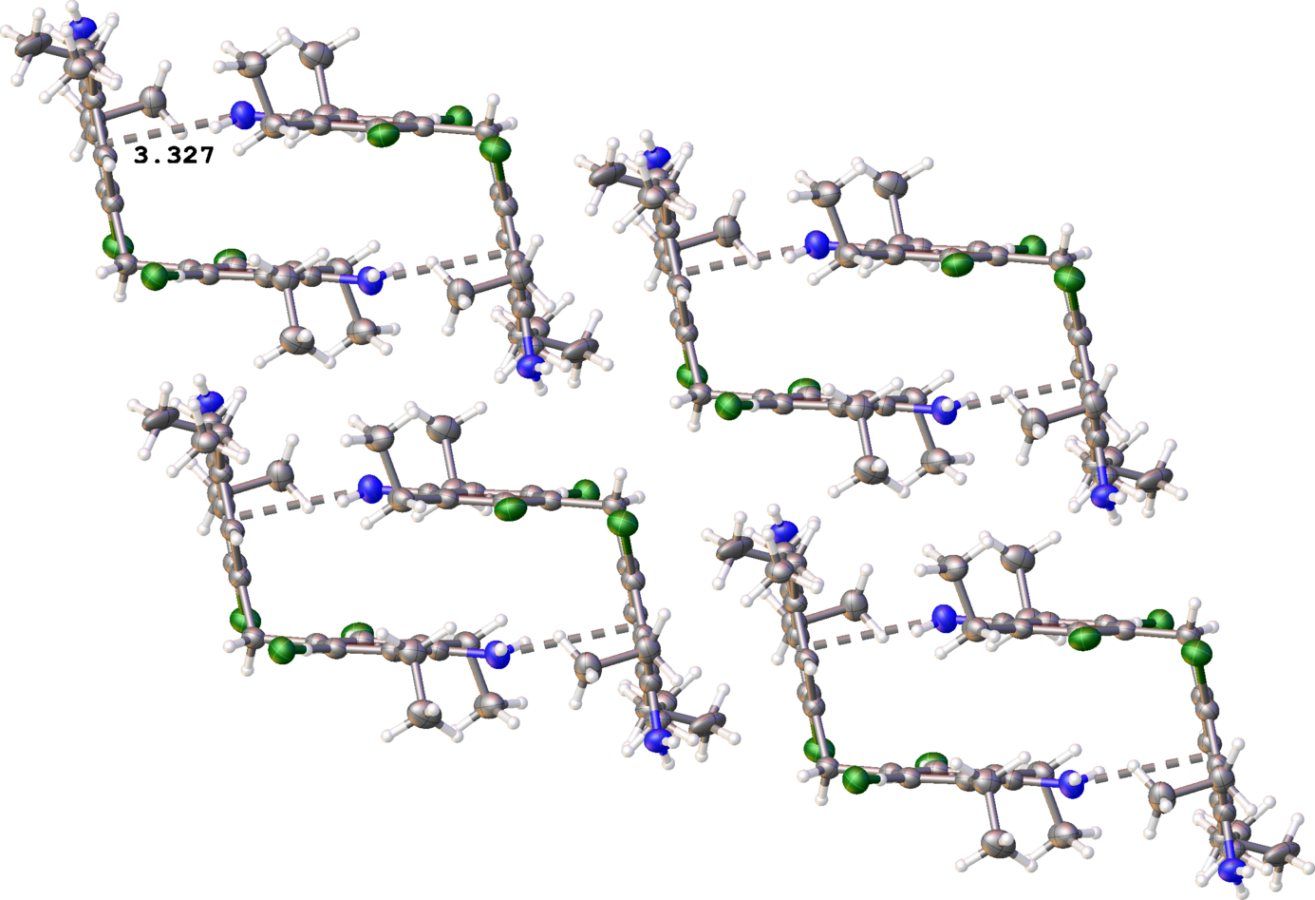
Fragment of the crystal packing of **2**.

In contrast, these types of inter­action are absent in the crystal structure of **2**. The mol­ecules form centrosymmetric dimers by N—H⋯π inter­actions [3.327 (2) Å] between N2—H2*A* and the C1–C6 ring centroid (Table 2 ▸, Fig. 4 ▸). Cohesion of the packing is provided by C7—H7*B*⋯π [3.579 (2) Å] and C20—H20*B*⋯π [3.434 (2) Å] inter­actions in the [100] direction and weak van der Waals inter­actions between the dimers.

## 4. Database survey

A search of the Cambridge Structural Database (CSD, version 5.45 updated to November 2023; Groom *et al.*, 2016 ▸) for **1** and **2** revealed that these structures had not been published previously. However, a similar uncharged structure without substituents in the ring, 4,4′-methyl­enebis(aniline), had been described in two independent experiments [CSD refcodes CEHCOH (Bel’skii *et al.*, 1983 ▸) and CEHCOH01 (Gibson *et al.*, 2010 ▸)]. Despite the similarity in mol­ecular structures and number of analogues inter­actions between these and the current study, such a herringbone-like packing motif does not occur in the previously published structures that can be explained by the rotational degree of freedom.

For the closest analogue with methyl substituents in the phenyl ring, 4,4′-methyl­enebis(2,6-di­methyl­aniline), a search resulted in 14 hits with CSD refcodes AWAYAZ–AWAYAZ13 (Bhattacharya & Saha, 2011 ▸). According to X-ray investigations, this structure was discovered in two polymorphic modifications in different space groups, *C*2/*c* and *P*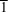; additionally, it was established in the original work that both polymorphs crystallize simultaneously from the solution in the presence of additional reagents. In general, the weakness of inter­molecular inter­actions is proved by the discovery of weak N—H⋯N inter­actions in one polymorphic form and the absence of such inter­actions in other structures.

The comparison of structures **1** and **2** and previously published analogues revealed that the twist angle between the two aromatic parts for the structure **1** [115.87 (6)°] lies within the characteristic range [44.21 (6)–134.35 (5)°], while the corresponding angle for the structure of **2** is smaller at 39.59 (8)°.

## 5. Synthesis and crystallization

The title compounds were prepared as follows:

**4,4′-Methyl­enebis(2,6-di­ethyl­aniline) (1)**

A mixture of 2,6-di­ethyl­aniline (14.92 g, 0.1 mol), paraformaldehyde (0.15 g, 0.05 mol) and 36% hydro­chloric acid (8.6 mL, 0.1 mol) in water (100 mL) in a round-bottom flask was heated to 353 K for 3 h in an oil bath under argon. The reaction mixture was cooled to room temperature and sodium hydroxide (4.40 g, 0.11 mol) was added. The precipitate was filtered and dried at 343 K in an oven in air for 12 h. Yield 14.52 g (94%). Single crystals suitable for X-ray analysis were grown by slow cooling (363–;303 K, 5 K h^−1^) of a solution of the substance in a DMSO/water (80:20, *v*:*v*) mixture.

^1^H NMR (600 MHz, DMSO-*d*_6_) δ 6.64 (*s*, 4H), 4.29 (*s*, 4H), 3.56 (*s*, 2H), 2.42 (*q*, *J* = 7.5 Hz, 8H), 1.09 (*t*, *J* = 7.5 Hz, 12H)^.^

**4,4′-Methyl­enebis(3-chloro-2,6-di­ethyl­aniline) (2)**

A mixture of 3-chloro-2,6-di­ethyl­aniline (18.38 g, 0.1 mol), paraformaldehyde (0.15 g, 0.05 mol) and 36% hydro­chloric acid (8.6 mL, 0.1 mol) in water (130 mL) in round-bottom flask was heated to 353 K for 3 h in an oil bath under argon. The reaction mixture was cooled to room temperature and sodium hydroxide (4.40 g, 0.11 mol) was added. The precipitate was filtered and dried at 343 K in an oven in air for 12 h. Yield 17.55 g (93%). Single crystals suitable for X-ray analysis were grown by slow evaporation of the solvent from a solution of the substance in toluene.

^1^H NMR (600 MHz, DMSO-*d*_6_) δ 6.51 (*s*, 2H), 4.70 (*s*, 4H), 3.84 (*s*, 2H), 2.72 (*q*, *J* = 7.4 Hz, 4H), 2.37 (*q*, *J* = 7.5 Hz, 4H), 1.08–0.99 (*m*, 12H).

## 6. Refinement

Crystal data, data collection and structure refinement details are summarized in Table 3 ▸. X-ray diffraction studies for (**2**) were carried out on the Belok’beamline (Svetogorov *et al.*, 2020 ▸) of the National Research Center "Kurchatov Institute" (Moscow, Russian Federation) using a Rayonix SX165 CCD detector. All hydrogen atoms in the structures of **1** and **2** were placed in calculated positions and refined using a riding model [*U*_iso_(H) = 1.2–1.5*U*_eq_(parent atom)]. In the structure of **2**, one chlorine atom was found to be disordered over two positions with a refined occupancy ratio of 0.920 (2): 0.080 (2). The ethyl group connected to the C2 atom was found to be disordered with occupancy ratios of 0.736 (11)/0.264 (11) in the structure of **1.** A SADI instruction was used to restrain the C7—C2 and C7′—C2 bonds in **1**.

## Supplementary Material

Crystal structure: contains datablock(s) 1, 2. DOI: 10.1107/S2056989025000234/ny2009sup1.cif

Structure factors: contains datablock(s) 1. DOI: 10.1107/S2056989025000234/ny20091sup2.hkl

Supporting information file. DOI: 10.1107/S2056989025000234/ny20091sup4.mol

Structure factors: contains datablock(s) 2. DOI: 10.1107/S2056989025000234/ny20092sup3.hkl

Supporting information file. DOI: 10.1107/S2056989025000234/ny20092sup5.mol

Supporting information file. DOI: 10.1107/S2056989025000234/ny20091sup6.cml

Supporting information file. DOI: 10.1107/S2056989025000234/ny20092sup7.cml

CCDC references: 2391630, 2391631

Additional supporting information:  crystallographic information; 3D view; checkCIF report

## Figures and Tables

**Figure 2:**
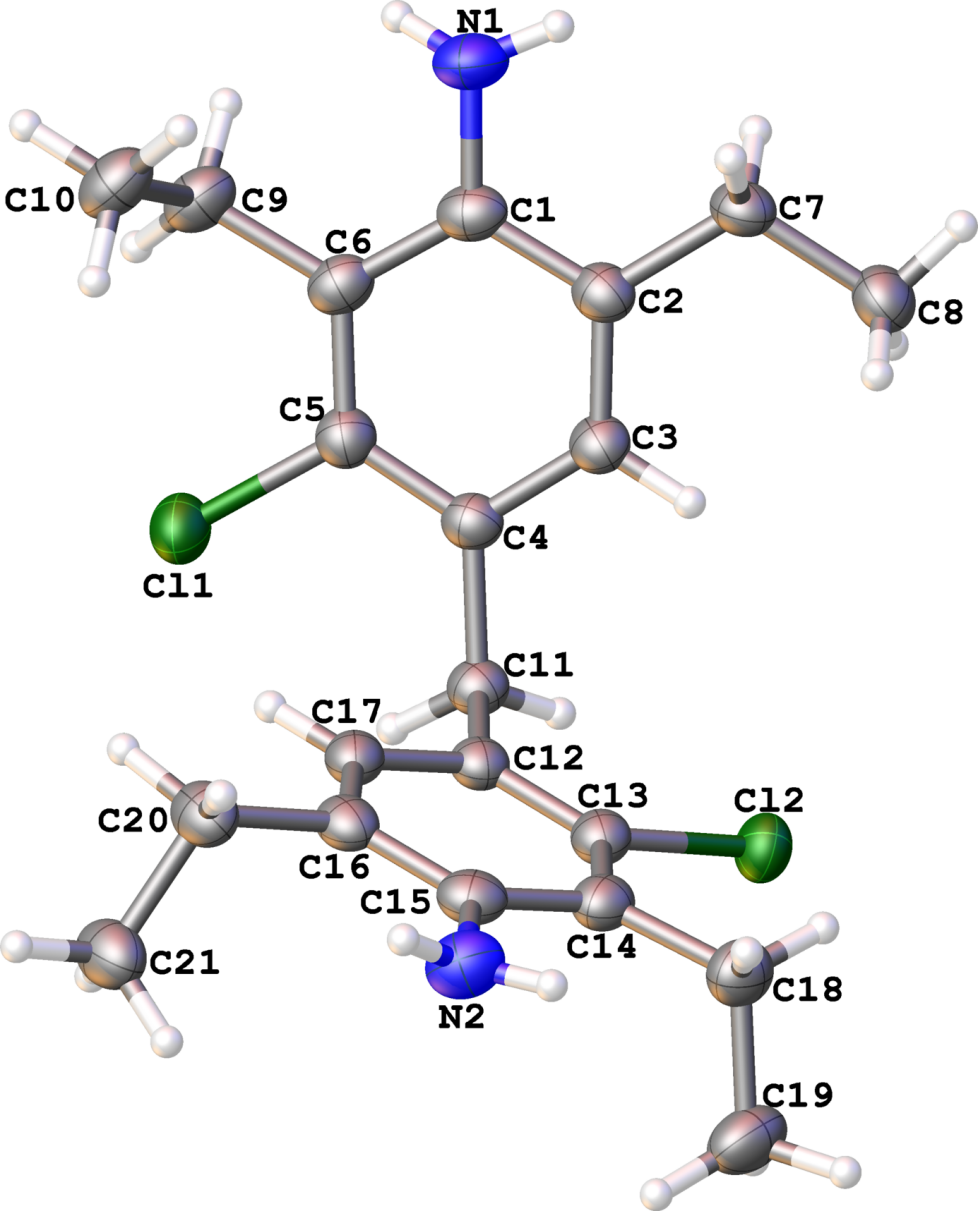
The mol­ecular structure of **2**, with displacement ellipsoids drawn at the 50% probability level. The minor occupancy components are omitted for clarity.

**Table 1:** Hydrogen-bond geometry (Å, °) for **1**[Chem scheme1] *Cg*1 is the centroid of the C1–C6 ring.

*D*—H⋯*A*	*D*—H	H⋯*A*	*D*⋯*A*	*D*—H⋯*A*
N2—H2*A*⋯N1^i^	0.88	2.62	3.472 (2)	165
C17—H17⋯*Cg*1^ii^	0.95	2.92	3.8568 (17)	171

Symmetry codes: (i) 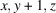; (ii) 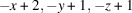.

**Table 2:** Hydrogen-bond geometry (Å, °) for **2**[Chem scheme1] *Cg*1 and *Cg*2 are the centroids of the C1–C6 and C12–C17 rings, respectively.

*D*—H⋯*A*	*D*—H	H⋯*A*	*D*⋯*A*	*D*—H⋯*A*
N2—H2*A*⋯*Cg*1^i^	0.88	2.63	3.327 (2)	137
C7—H7*B*⋯*Cg*2^ii^	0.99	2.83	3.579 (2)	133
C20—H20*B*⋯*Cg*2^i^	0.99	2.81	3.434 (2)	132

Symmetry codes: (i) 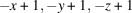; (ii) 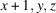.

**Table 3:** Experimental details

	**1**	**2**
Crystal data		
Chemical formula	C_21_H_30_N_2_	C_21_H_28_Cl_2_N_2_
*M*_r_	310.47	379.35
Crystal system, space group	Monoclinic, *P*2_1_/*n*	Triclinic, *P* 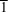
Temperature (K)	150	100
*a*, *b*, *c* (Å)	8.9895 (3), 11.8589 (3), 17.6765 (5)	8.993 (3), 9.6540 (13), 12.216 (4)
α, β, γ (°)	90, 102.4188 (11), 90	69.352 (11), 77.257 (12), 87.670 (8)
*V* (Å^3^)	1840.32 (9)	967.2 (5)
*Z*	4	2
Radiation type	Cu *K*α	Synchrotron, λ = 0.75268 Å
μ (mm^−1^)	0.49	0.40
Crystal size (mm)	0.32 × 0.21 × 0.13	0.19 × 0.12 × 0.05

Data collection		
Diffractometer	Bruker D8 Venture	Rayonix SX165 CCD
Absorption correction	Multi-scan (*SADABS*; Krause *et al.*, 2015 ▸)	Empirical (using intensity measurements) [*XDS* (Kabsch, 2010 ▸)]
*T*_min_, *T*_max_	0.639, 0.753	0.001, 1.000
No. of measured, independent and observed [*I* > 2σ(*I*)] reflections	22931, 3455, 3212	17607, 4844, 3879
*R*_int_	0.035	0.053
(sin θ/λ)_max_ (Å^−1^)	0.608	0.675

Refinement		
*R*[*F*^2^ > 2σ(*F*^2^)], *wR*(*F*^2^), *S*	0.050, 0.135, 1.03	0.051, 0.147, 1.05
No. of reflections	3455	4844
No. of parameters	236	243
No. of restraints	1	0
H-atom treatment	H-atom parameters constrained	H-atom parameters constrained
Δρ_max_, Δρ_min_ (e Å^−3^)	0.51, −0.33	0.64, −0.56

Computer programs: *APEX3* v.2017.3_0 (Bruker, 2016 ▸), *Marccd* (Doyle, 2011 ▸), *XDS* (Kabsch, 2010 ▸), *SAINT* (Bruker, 2016 ▸), *SHELXT2014/5* (Sheldrick, 2015*a* ▸), *SHELXL2019/3* (Sheldrick, 2015*b* ▸), *OLEX2* 1.3 (Dolomanov *et al.*, 2009 ▸).

## supplementary crystallographic information

### 4,4'-(Methanediyl)bis(2,6-diethylaniline) (1) Crystal data

**Table:**

C_21_H_30_N_2_	*F*(000) = 680
*M**_r_* = 310.47	*D*_x_ = 1.121 Mg m^−^^3^
Monoclinic, *P*2_1_/*n*	Cu *K*α radiation, λ = 1.54178 Å
*a* = 8.9895 (3) Å	Cell parameters from 9874 reflections
*b* = 11.8589 (3) Å	θ = 4.5–69.6°
*c* = 17.6765 (5) Å	µ = 0.49 mm^−^^1^
β = 102.4188 (11)°	*T* = 150 K
*V* = 1840.32 (9) Å^3^	Prism, colourless
*Z* = 4	0.32 × 0.21 × 0.13 mm

### 4,4'-(Methanediyl)bis(2,6-diethylaniline) (1) Data collection

**Table:**

Bruker D8 Venture diffractometer	3212 reflections with *I* > 2σ(*I*)
Radiation source: microfocus sealed X-ray tube	*R*_int_ = 0.035
φ and ω scans	θ_max_ = 69.6°, θ_min_ = 4.5°
Absorption correction: multi-scan (SADABS; Krause *et al.*, 2015)	*h* = −10→10
*T*_min_ = 0.639, *T*_max_ = 0.753	*k* = −14→14
22931 measured reflections	*l* = −21→21
3455 independent reflections	

### 4,4'-(Methanediyl)bis(2,6-diethylaniline) (1) Refinement

**Table:**

Refinement on *F*^2^	Primary atom site location: structure-invariant direct methods
Least-squares matrix: full	Secondary atom site location: difference Fourier map
*R*[*F*^2^ > 2σ(*F*^2^)] = 0.050	H-atom parameters constrained
*wR*(*F*^2^) = 0.135	*w* = 1/[σ^2^(*F*_o_^2^) + (0.0668*P*)^2^ + 0.8039*P*] where *P* = (*F*_o_^2^ + 2*F*_c_^2^)/3
*S* = 1.03	(Δ/σ)_max_ < 0.001
3455 reflections	Δρ_max_ = 0.51 e Å^−^^3^
236 parameters	Δρ_min_ = −0.33 e Å^−^^3^
1 restraint	

### 4,4'-(Methanediyl)bis(2,6-diethylaniline) (1) Special details

**Table:**

Geometry. All esds (except the esd in the dihedral angle between two l.s. planes) are estimated using the full covariance matrix. The cell esds are taken into account individually in the estimation of esds in distances, angles and torsion angles; correlations between esds in cell parameters are only used when they are defined by crystal symmetry. An approximate (isotropic) treatment of cell esds is used for estimating esds involving l.s. planes.

### 4,4'-(Methanediyl)bis(2,6-diethylaniline) (1) Fractional atomic coordinates and isotropic or equivalent isotropic displacement parameters (Å^2^)

**Table:**

	*x*	*y*	*z*	*U*_iso_*/*U*_eq_	Occ. (<1)
N1	0.50801 (18)	0.22887 (12)	0.44320 (9)	0.0495 (4)	
H1A	0.444518	0.203843	0.401577	0.073 (7)*	
H1B	0.454778	0.243338	0.478419	0.042 (5)*	
N2	0.76330 (16)	1.02220 (12)	0.52830 (8)	0.0434 (3)	
H2A	0.702084	1.069094	0.497865	0.052*	
H2B	0.841893	1.056893	0.557216	0.052*	
C1	0.59366 (18)	0.32315 (12)	0.42982 (9)	0.0371 (3)	
C2	0.6281 (2)	0.34245 (13)	0.35674 (9)	0.0450 (4)	
C3	0.7176 (2)	0.43384 (13)	0.34743 (8)	0.0422 (4)	
H3	0.738899	0.447362	0.297852	0.051*	
C4	0.77715 (16)	0.50617 (12)	0.40722 (8)	0.0322 (3)	
C5	0.74348 (15)	0.48530 (12)	0.47942 (8)	0.0288 (3)	
H5	0.782842	0.534865	0.521151	0.035*	
C6	0.65472 (15)	0.39491 (11)	0.49237 (8)	0.0293 (3)	
C7	0.5780 (5)	0.2673 (2)	0.28671 (14)	0.0371 (8)	0.736 (11)
H7A	0.572874	0.188130	0.303718	0.045*	0.736 (11)
H7B	0.653349	0.271536	0.253388	0.045*	0.736 (11)
C7'	0.493 (2)	0.2769 (8)	0.2921 (5)	0.056 (4)	0.264 (11)
H7'A	0.522766	0.196888	0.288739	0.068*	0.264 (11)
H7'B	0.396857	0.278633	0.310534	0.068*	0.264 (11)
C8	0.4216 (5)	0.3041 (3)	0.2405 (3)	0.0527 (11)	0.736 (11)
H8A	0.391557	0.255842	0.194700	0.079*	0.736 (11)
H8B	0.426673	0.382675	0.224074	0.079*	0.736 (11)
H8C	0.346445	0.297264	0.272966	0.079*	0.736 (11)
C8'	0.4671 (12)	0.3274 (9)	0.2140 (6)	0.047 (2)	0.264 (11)
H8'A	0.403094	0.276881	0.176766	0.070*	0.264 (11)
H8'B	0.564933	0.338586	0.199170	0.070*	0.264 (11)
H8'C	0.415683	0.400219	0.214345	0.070*	0.264 (11)
C9	0.62289 (17)	0.36943 (13)	0.57138 (8)	0.0355 (3)	
H9A	0.659524	0.292198	0.586402	0.043*	
H9B	0.511194	0.369814	0.566853	0.043*	
C10	0.6940 (2)	0.44930 (16)	0.63601 (9)	0.0455 (4)	
H10A	0.659453	0.526391	0.622011	0.068*	
H10B	0.805186	0.445821	0.643944	0.068*	
H10C	0.663280	0.427331	0.683880	0.068*	
C11	0.88289 (18)	0.60062 (13)	0.39550 (9)	0.0391 (4)	
H11A	0.988886	0.577452	0.418229	0.047*	
H11B	0.874584	0.611130	0.339183	0.047*	
C12	0.85318 (16)	0.71251 (12)	0.43033 (8)	0.0339 (3)	
C13	0.73112 (15)	0.78002 (12)	0.39480 (8)	0.0314 (3)	
H13	0.666330	0.754365	0.348255	0.038*	
C14	0.70101 (16)	0.88340 (12)	0.42509 (8)	0.0317 (3)	
C15	0.79580 (17)	0.92047 (13)	0.49506 (8)	0.0342 (3)	
C16	0.91879 (17)	0.85307 (14)	0.53197 (8)	0.0373 (3)	
C17	0.94554 (16)	0.75095 (14)	0.49856 (9)	0.0374 (3)	
H17	1.029684	0.706187	0.523247	0.045*	
C18	0.57200 (17)	0.95838 (13)	0.38505 (9)	0.0385 (3)	
H18A	0.615497	1.031988	0.374583	0.046*	
H18B	0.504104	0.972245	0.421362	0.046*	
C19	0.47564 (19)	0.91440 (15)	0.30954 (10)	0.0464 (4)	
H19A	0.539667	0.904872	0.271582	0.070*	
H19B	0.394034	0.968336	0.289597	0.070*	
H19C	0.431041	0.841598	0.318726	0.070*	
C20	1.0202 (2)	0.88664 (17)	0.60861 (9)	0.0477 (4)	
H20A	1.119249	0.847402	0.614784	0.057*	
H20B	1.039622	0.968796	0.608464	0.057*	
C21	0.9492 (3)	0.8578 (2)	0.67641 (11)	0.0614 (5)	
H21A	0.852117	0.897807	0.671087	0.092*	
H21B	1.018192	0.880692	0.724765	0.092*	
H21C	0.931458	0.776368	0.677302	0.092*	

### 4,4'-(Methanediyl)bis(2,6-diethylaniline) (1) Atomic displacement parameters (Å^2^)

**Table:**

	*U*^11^	*U*^22^	*U*^33^	*U*^12^	*U*^13^	*U*^23^
N1	0.0589 (9)	0.0356 (7)	0.0489 (8)	−0.0072 (6)	0.0002 (7)	0.0029 (6)
N2	0.0480 (8)	0.0408 (7)	0.0411 (7)	−0.0051 (6)	0.0087 (6)	−0.0037 (6)
C1	0.0444 (8)	0.0276 (7)	0.0353 (8)	0.0053 (6)	0.0000 (6)	0.0041 (6)
C2	0.0686 (11)	0.0323 (8)	0.0296 (8)	0.0050 (7)	0.0005 (7)	−0.0005 (6)
C3	0.0659 (10)	0.0359 (8)	0.0257 (7)	0.0120 (7)	0.0121 (7)	0.0035 (6)
C4	0.0348 (7)	0.0317 (7)	0.0309 (7)	0.0117 (6)	0.0088 (6)	0.0056 (5)
C5	0.0284 (6)	0.0307 (7)	0.0264 (6)	0.0086 (5)	0.0037 (5)	0.0006 (5)
C6	0.0293 (7)	0.0300 (7)	0.0277 (7)	0.0101 (5)	0.0044 (5)	0.0036 (5)
C7	0.0406 (18)	0.0373 (12)	0.0329 (12)	0.0057 (11)	0.0069 (10)	−0.0055 (8)
C7'	0.089 (10)	0.039 (4)	0.037 (4)	−0.018 (5)	0.003 (5)	−0.003 (3)
C8	0.056 (2)	0.0475 (17)	0.045 (2)	0.0010 (14)	−0.0112 (16)	−0.0127 (15)
C8'	0.050 (5)	0.049 (4)	0.041 (5)	−0.003 (4)	0.007 (3)	−0.007 (4)
C9	0.0344 (7)	0.0403 (8)	0.0328 (7)	0.0075 (6)	0.0099 (6)	0.0074 (6)
C10	0.0533 (9)	0.0575 (10)	0.0291 (7)	0.0086 (8)	0.0163 (7)	−0.0005 (7)
C11	0.0380 (8)	0.0419 (8)	0.0403 (8)	0.0090 (6)	0.0151 (6)	0.0086 (6)
C12	0.0308 (7)	0.0361 (8)	0.0367 (7)	0.0000 (6)	0.0114 (6)	0.0091 (6)
C13	0.0295 (7)	0.0355 (7)	0.0292 (7)	−0.0026 (5)	0.0065 (5)	0.0048 (5)
C14	0.0302 (7)	0.0339 (7)	0.0316 (7)	−0.0024 (6)	0.0081 (5)	0.0067 (6)
C15	0.0368 (7)	0.0351 (7)	0.0325 (7)	−0.0064 (6)	0.0112 (6)	0.0036 (6)
C16	0.0334 (7)	0.0455 (9)	0.0322 (7)	−0.0071 (6)	0.0053 (6)	0.0088 (6)
C17	0.0304 (7)	0.0434 (8)	0.0373 (8)	0.0004 (6)	0.0050 (6)	0.0128 (6)
C18	0.0373 (8)	0.0365 (8)	0.0411 (8)	0.0042 (6)	0.0073 (6)	0.0052 (6)
C19	0.0415 (8)	0.0498 (9)	0.0434 (9)	0.0082 (7)	−0.0010 (7)	0.0077 (7)
C20	0.0445 (9)	0.0587 (10)	0.0363 (8)	−0.0091 (8)	0.0009 (7)	0.0062 (7)
C21	0.0733 (13)	0.0726 (13)	0.0371 (9)	−0.0096 (11)	0.0091 (9)	0.0061 (9)

### 4,4'-(Methanediyl)bis(2,6-diethylaniline) (1) Geometric parameters (Å, º)

**Table:**

N1—C1	1.406 (2)	C9—H9A	0.9900
N1—H1A	0.8801	C9—H9B	0.9900
N1—H1B	0.8799	C10—H10A	0.9800
N2—C15	1.400 (2)	C10—H10B	0.9800
N2—H2A	0.8799	C10—H10C	0.9800
N2—H2B	0.8799	C11—C12	1.511 (2)
C1—C6	1.410 (2)	C11—H11A	0.9900
C1—C2	1.410 (2)	C11—H11B	0.9900
C2—C3	1.380 (2)	C12—C17	1.386 (2)
C2—C7	1.513 (3)	C12—C13	1.394 (2)
C2—C7'	1.668 (9)	C13—C14	1.388 (2)
C3—C4	1.377 (2)	C13—H13	0.9500
C3—H3	0.9500	C14—C15	1.413 (2)
C4—C5	1.3957 (19)	C14—C18	1.511 (2)
C4—C11	1.512 (2)	C15—C16	1.406 (2)
C5—C6	1.384 (2)	C16—C17	1.391 (2)
C5—H5	0.9500	C16—C20	1.514 (2)
C6—C9	1.5151 (19)	C17—H17	0.9500
C7—C8	1.530 (5)	C18—C19	1.519 (2)
C7—H7A	0.9900	C18—H18A	0.9900
C7—H7B	0.9900	C18—H18B	0.9900
C7'—C8'	1.476 (15)	C19—H19A	0.9800
C7'—H7'A	0.9900	C19—H19B	0.9800
C7'—H7'B	0.9900	C19—H19C	0.9800
C8—H8A	0.9800	C20—C21	1.513 (2)
C8—H8B	0.9800	C20—H20A	0.9900
C8—H8C	0.9800	C20—H20B	0.9900
C8'—H8'A	0.9800	C21—H21A	0.9800
C8'—H8'B	0.9800	C21—H21B	0.9800
C8'—H8'C	0.9800	C21—H21C	0.9800
C9—C10	1.516 (2)		
			
C1—N1—H1A	114.1	H9A—C9—H9B	107.4
C1—N1—H1B	111.4	C9—C10—H10A	109.5
H1A—N1—H1B	107.7	C9—C10—H10B	109.5
C15—N2—H2A	116.6	H10A—C10—H10B	109.5
C15—N2—H2B	115.6	C9—C10—H10C	109.5
H2A—N2—H2B	112.2	H10A—C10—H10C	109.5
N1—C1—C6	118.89 (14)	H10B—C10—H10C	109.5
N1—C1—C2	121.28 (14)	C12—C11—C4	115.05 (12)
C6—C1—C2	119.68 (14)	C12—C11—H11A	108.5
C3—C2—C1	119.02 (14)	C4—C11—H11A	108.5
C3—C2—C7	116.33 (19)	C12—C11—H11B	108.5
C1—C2—C7	124.6 (2)	C4—C11—H11B	108.5
C3—C2—C7'	129.8 (3)	H11A—C11—H11B	107.5
C1—C2—C7'	106.3 (5)	C17—C12—C13	117.99 (14)
C4—C3—C2	122.54 (14)	C17—C12—C11	121.21 (13)
C4—C3—H3	118.7	C13—C12—C11	120.79 (13)
C2—C3—H3	118.7	C14—C13—C12	122.25 (13)
C3—C4—C5	117.81 (14)	C14—C13—H13	118.9
C3—C4—C11	120.56 (13)	C12—C13—H13	118.9
C5—C4—C11	121.54 (13)	C13—C14—C15	118.80 (13)
C6—C5—C4	122.31 (13)	C13—C14—C18	122.21 (13)
C6—C5—H5	118.8	C15—C14—C18	118.99 (13)
C4—C5—H5	118.8	N2—C15—C16	120.62 (14)
C5—C6—C1	118.61 (13)	N2—C15—C14	119.65 (14)
C5—C6—C9	122.46 (13)	C16—C15—C14	119.70 (14)
C1—C6—C9	118.93 (13)	C17—C16—C15	119.23 (14)
C2—C7—C8	110.3 (3)	C17—C16—C20	118.89 (15)
C2—C7—H7A	109.6	C15—C16—C20	121.84 (15)
C8—C7—H7A	109.6	C12—C17—C16	122.01 (14)
C2—C7—H7B	109.6	C12—C17—H17	119.0
C8—C7—H7B	109.6	C16—C17—H17	119.0
H7A—C7—H7B	108.1	C14—C18—C19	116.41 (13)
C8'—C7'—C2	113.4 (8)	C14—C18—H18A	108.2
C8'—C7'—H7'A	108.9	C19—C18—H18A	108.2
C2—C7'—H7'A	108.9	C14—C18—H18B	108.2
C8'—C7'—H7'B	108.9	C19—C18—H18B	108.2
C2—C7'—H7'B	108.9	H18A—C18—H18B	107.3
H7'A—C7'—H7'B	107.7	C18—C19—H19A	109.5
C7—C8—H8A	109.5	C18—C19—H19B	109.5
C7—C8—H8B	109.5	H19A—C19—H19B	109.5
H8A—C8—H8B	109.5	C18—C19—H19C	109.5
C7—C8—H8C	109.5	H19A—C19—H19C	109.5
H8A—C8—H8C	109.5	H19B—C19—H19C	109.5
H8B—C8—H8C	109.5	C21—C20—C16	111.99 (15)
C7'—C8'—H8'A	109.5	C21—C20—H20A	109.2
C7'—C8'—H8'B	109.5	C16—C20—H20A	109.2
H8'A—C8'—H8'B	109.5	C21—C20—H20B	109.2
C7'—C8'—H8'C	109.5	C16—C20—H20B	109.2
H8'A—C8'—H8'C	109.5	H20A—C20—H20B	107.9
H8'B—C8'—H8'C	109.5	C20—C21—H21A	109.5
C6—C9—C10	115.95 (13)	C20—C21—H21B	109.5
C6—C9—H9A	108.3	H21A—C21—H21B	109.5
C10—C9—H9A	108.3	C20—C21—H21C	109.5
C6—C9—H9B	108.3	H21A—C21—H21C	109.5
C10—C9—H9B	108.3	H21B—C21—H21C	109.5
			
N1—C1—C2—C3	−177.50 (15)	C3—C4—C11—C12	137.06 (15)
C6—C1—C2—C3	−2.0 (2)	C5—C4—C11—C12	−46.59 (19)
N1—C1—C2—C7	0.4 (3)	C4—C11—C12—C17	103.56 (16)
C6—C1—C2—C7	175.9 (2)	C4—C11—C12—C13	−76.11 (17)
N1—C1—C2—C7'	24.7 (6)	C17—C12—C13—C14	0.6 (2)
C6—C1—C2—C7'	−159.8 (6)	C11—C12—C13—C14	−179.74 (12)
C1—C2—C3—C4	1.2 (3)	C12—C13—C14—C15	−1.2 (2)
C7—C2—C3—C4	−176.90 (19)	C12—C13—C14—C18	177.80 (13)
C7'—C2—C3—C4	153.0 (9)	C13—C14—C15—N2	−177.22 (12)
C2—C3—C4—C5	−0.5 (2)	C18—C14—C15—N2	3.8 (2)
C2—C3—C4—C11	175.98 (15)	C13—C14—C15—C16	0.7 (2)
C3—C4—C5—C6	0.6 (2)	C18—C14—C15—C16	−178.32 (13)
C11—C4—C5—C6	−175.81 (12)	N2—C15—C16—C17	178.22 (13)
C4—C5—C6—C1	−1.5 (2)	C14—C15—C16—C17	0.4 (2)
C4—C5—C6—C9	177.61 (12)	N2—C15—C16—C20	0.3 (2)
N1—C1—C6—C5	177.72 (13)	C14—C15—C16—C20	−177.59 (13)
C2—C1—C6—C5	2.1 (2)	C13—C12—C17—C16	0.5 (2)
N1—C1—C6—C9	−1.4 (2)	C11—C12—C17—C16	−179.18 (13)
C2—C1—C6—C9	−176.97 (13)	C15—C16—C17—C12	−1.0 (2)
C3—C2—C7—C8	−93.4 (5)	C20—C16—C17—C12	177.05 (14)
C1—C2—C7—C8	88.6 (4)	C13—C14—C18—C19	0.3 (2)
C3—C2—C7'—C8'	−1.0 (18)	C15—C14—C18—C19	179.22 (13)
C1—C2—C7'—C8'	153.5 (12)	C17—C16—C20—C21	−97.07 (19)
C5—C6—C9—C10	0.1 (2)	C15—C16—C20—C21	80.9 (2)
C1—C6—C9—C10	179.21 (13)		

### 4,4'-(Methanediyl)bis(2,6-diethylaniline) (1) Hydrogen-bond geometry (Å, º)

*Cg*1 is the centroid of the C1–C6 ring.

**Table:**

*D*—H···*A*	*D*—H	H···*A*	*D*···*A*	*D*—H···*A*
N2—H2*A*···N1^i^	0.88	2.62	3.472 (2)	165
C17—H17···*Cg*1^ii^	0.95	2.92	3.8568 (17)	171

Symmetry codes: (i) *x*, *y*+1, *z*; (ii) −*x*+2, −*y*+1, −*z*+1.

### 4,4'-(MethAnediyl)bis(3-chloro-2,6-diethylaniline) (2) Crystal data

**Table:**

C_21_H_28_Cl_2_N_2_	*Z* = 2
*M**_r_* = 379.35	*F*(000) = 404
Triclinic, *P*1	*D*_x_ = 1.303 Mg m^−^^3^
*a* = 8.993 (3) Å	Synchrotron radiation, λ = 0.75268 Å
*b* = 9.6540 (13) Å	Cell parameters from 5979 reflections
*c* = 12.216 (4) Å	θ = 1.7–31.0°
α = 69.352 (11)°	µ = 0.40 mm^−^^1^
β = 77.257 (12)°	*T* = 100 K
γ = 87.670 (8)°	Plate, colourless
*V* = 967.2 (5) Å^3^	0.19 × 0.12 × 0.05 mm

### 4,4'-(MethAnediyl)bis(3-chloro-2,6-diethylaniline) (2) Data collection

**Table:**

Rayonix SX165 CCD diffractometer	3879 reflections with *I* > 2σ(*I*)
φ scans	*R*_int_ = 0.053
Absorption correction: empirical (using intensity measurements) [*XDS* (Kabsch, 2010)]	θ_max_ = 30.5°, θ_min_ = 2.4°
*T*_min_ = 0.001, *T*_max_ = 1.000	*h* = −12→12
17607 measured reflections	*k* = −13→13
4844 independent reflections	*l* = −16→16

### 4,4'-(MethAnediyl)bis(3-chloro-2,6-diethylaniline) (2) Refinement

**Table:**

Refinement on *F*^2^	Secondary atom site location: difference Fourier map
Least-squares matrix: full	H-atom parameters constrained
*R*[*F*^2^ > 2σ(*F*^2^)] = 0.051	*w* = 1/[σ^2^(*F*_o_^2^) + (0.0847*P*)^2^ + 0.2296*P*] where *P* = (*F*_o_^2^ + 2*F*_c_^2^)/3
*wR*(*F*^2^) = 0.147	(Δ/σ)_max_ = 0.001
*S* = 1.05	Δρ_max_ = 0.64 e Å^−^^3^
4844 reflections	Δρ_min_ = −0.56 e Å^−^^3^
243 parameters	Extinction correction: SHELXL-2019/2 (Sheldrick 2015b), Fc^*^=kFc[1+0.001xFc^2^λ^3^/sin(2θ)]^-1/4^
0 restraints	Extinction coefficient: 0.090 (12)
Primary atom site location: structure-invariant direct methods	

### 4,4'-(MethAnediyl)bis(3-chloro-2,6-diethylaniline) (2) Special details

**Table:**

Geometry. All esds (except the esd in the dihedral angle between two l.s. planes) are estimated using the full covariance matrix. The cell esds are taken into account individually in the estimation of esds in distances, angles and torsion angles; correlations between esds in cell parameters are only used when they are defined by crystal symmetry. An approximate (isotropic) treatment of cell esds is used for estimating esds involving l.s. planes.

### 4,4'-(MethAnediyl)bis(3-chloro-2,6-diethylaniline) (2) Fractional atomic coordinates and isotropic or equivalent isotropic displacement parameters (Å^2^)

**Table:**

	*x*	*y*	*z*	*U*_iso_*/*U*_eq_	Occ. (<1)
Cl1	0.60707 (6)	1.01312 (5)	0.11839 (5)	0.04260 (17)	
Cl2	0.57096 (6)	0.36463 (6)	0.14187 (5)	0.03812 (19)	0.920 (2)
Cl2'	0.4357 (7)	0.8207 (7)	0.3210 (6)	0.0426 (19)	0.080 (2)
N1	1.1478 (2)	0.8869 (2)	0.18791 (17)	0.0425 (4)	
H1A	1.162855	0.983508	0.163730	0.051*	
H1B	1.220754	0.824611	0.182099	0.051*	
N2	0.2511 (2)	0.3094 (2)	0.56093 (16)	0.0444 (4)	
H2A	0.232317	0.332820	0.625941	0.053*	
H2B	0.268619	0.216227	0.569476	0.053*	
C1	1.0157 (2)	0.8455 (2)	0.16291 (16)	0.0348 (4)	
C2	1.0027 (2)	0.7044 (2)	0.15586 (16)	0.0335 (4)	
C3	0.8665 (2)	0.6623 (2)	0.13837 (16)	0.0319 (4)	
H3	0.857145	0.566189	0.135205	0.038*	
C4	0.7421 (2)	0.7542 (2)	0.12515 (15)	0.0307 (3)	
C5	0.7613 (2)	0.8934 (2)	0.13066 (16)	0.0326 (4)	
C6	0.8947 (2)	0.9433 (2)	0.14874 (16)	0.0340 (4)	
C7	1.1390 (2)	0.6070 (2)	0.16344 (18)	0.0382 (4)	
H7A	1.229620	0.666353	0.106943	0.046*	
H7B	1.157666	0.578413	0.245303	0.046*	
C8	1.1257 (3)	0.4681 (3)	0.1365 (2)	0.0443 (5)	
H8A	1.105026	0.494073	0.056275	0.066*	
H8B	1.041972	0.403709	0.196063	0.066*	
H8C	1.221399	0.416063	0.139488	0.066*	
C9	0.9082 (3)	1.0940 (2)	0.15711 (18)	0.0405 (4)	
H9A	0.842955	1.162596	0.108485	0.049*	
H9B	1.015074	1.132801	0.123406	0.049*	
C10	0.8605 (3)	1.0892 (2)	0.28658 (19)	0.0459 (5)	
H10A	0.870197	1.189198	0.288216	0.069*	
H10B	0.926583	1.023404	0.334555	0.069*	
H10C	0.754312	1.052072	0.319926	0.069*	
C11	0.5968 (2)	0.6974 (2)	0.10932 (16)	0.0318 (4)	
H11A	0.622748	0.646743	0.050261	0.038*	
H11B	0.533729	0.782290	0.077199	0.038*	
C12	0.5054 (2)	0.5907 (2)	0.22658 (16)	0.0301 (3)	
C13	0.4873 (2)	0.4395 (2)	0.25223 (16)	0.0312 (4)	
H13	0.531639	0.400676	0.191570	0.037*	0.080 (2)
C14	0.4072 (2)	0.3412 (2)	0.36264 (17)	0.0343 (4)	
C15	0.3368 (2)	0.4002 (2)	0.45127 (16)	0.0345 (4)	
C16	0.3530 (2)	0.5527 (2)	0.42903 (17)	0.0338 (4)	
C17	0.4371 (2)	0.6427 (2)	0.31877 (16)	0.0316 (4)	
H17	0.449079	0.745242	0.304869	0.038*	0.920 (2)
C18	0.3987 (3)	0.1759 (2)	0.3901 (2)	0.0413 (4)	
H18A	0.403956	0.124330	0.474962	0.050*	
H18B	0.488723	0.148971	0.339552	0.050*	
C19	0.2542 (3)	0.1209 (3)	0.3690 (2)	0.0524 (6)	
H19A	0.250468	0.167582	0.284280	0.079*	
H19B	0.164323	0.146679	0.418874	0.079*	
H19C	0.255270	0.013057	0.390275	0.079*	
C20	0.2797 (2)	0.6213 (3)	0.52112 (19)	0.0410 (4)	
H20A	0.334078	0.716805	0.502372	0.049*	
H20B	0.291900	0.555093	0.601287	0.049*	
C21	0.1098 (3)	0.6484 (3)	0.5257 (2)	0.0481 (5)	
H21A	0.096024	0.709184	0.445546	0.072*	
H21B	0.071381	0.700143	0.581735	0.072*	
H21C	0.053358	0.553298	0.552671	0.072*	

### 4,4'-(MethAnediyl)bis(3-chloro-2,6-diethylaniline) (2) Atomic displacement parameters (Å^2^)

**Table:**

	*U*^11^	*U*^22^	*U*^33^	*U*^12^	*U*^13^	*U*^23^
Cl1	0.0462 (3)	0.0385 (3)	0.0499 (3)	0.0102 (2)	−0.0180 (2)	−0.0203 (2)
Cl2	0.0439 (3)	0.0365 (3)	0.0389 (3)	0.0056 (2)	−0.0091 (2)	−0.0197 (2)
Cl2'	0.035 (3)	0.045 (3)	0.058 (4)	0.005 (2)	−0.017 (3)	−0.027 (3)
N1	0.0361 (9)	0.0487 (10)	0.0464 (9)	−0.0057 (7)	−0.0128 (7)	−0.0182 (8)
N2	0.0486 (10)	0.0485 (10)	0.0345 (8)	−0.0073 (8)	−0.0085 (8)	−0.0122 (7)
C1	0.0338 (9)	0.0434 (10)	0.0289 (8)	−0.0044 (7)	−0.0075 (7)	−0.0137 (7)
C2	0.0320 (9)	0.0408 (9)	0.0305 (8)	0.0035 (7)	−0.0093 (7)	−0.0149 (7)
C3	0.0337 (9)	0.0362 (8)	0.0300 (8)	0.0027 (7)	−0.0103 (7)	−0.0148 (7)
C4	0.0298 (8)	0.0356 (8)	0.0274 (8)	0.0002 (7)	−0.0070 (7)	−0.0116 (7)
C5	0.0348 (9)	0.0340 (8)	0.0304 (8)	0.0029 (7)	−0.0092 (7)	−0.0120 (7)
C6	0.0381 (9)	0.0350 (9)	0.0293 (8)	−0.0039 (7)	−0.0073 (7)	−0.0114 (7)
C7	0.0323 (9)	0.0482 (10)	0.0388 (10)	0.0058 (8)	−0.0127 (8)	−0.0187 (8)
C8	0.0408 (10)	0.0486 (11)	0.0487 (11)	0.0104 (9)	−0.0149 (9)	−0.0216 (9)
C9	0.0483 (11)	0.0367 (9)	0.0371 (10)	−0.0071 (8)	−0.0092 (9)	−0.0132 (8)
C10	0.0581 (13)	0.0417 (10)	0.0409 (10)	−0.0098 (9)	−0.0078 (10)	−0.0187 (9)
C11	0.0316 (8)	0.0352 (8)	0.0312 (8)	0.0010 (7)	−0.0100 (7)	−0.0130 (7)
C12	0.0274 (8)	0.0348 (8)	0.0323 (8)	0.0031 (6)	−0.0115 (7)	−0.0142 (7)
C13	0.0305 (8)	0.0357 (8)	0.0324 (8)	0.0031 (7)	−0.0118 (7)	−0.0152 (7)
C14	0.0333 (9)	0.0349 (9)	0.0385 (9)	0.0022 (7)	−0.0145 (8)	−0.0137 (7)
C15	0.0304 (8)	0.0433 (10)	0.0310 (8)	−0.0009 (7)	−0.0117 (7)	−0.0115 (7)
C16	0.0297 (8)	0.0444 (10)	0.0340 (9)	0.0043 (7)	−0.0126 (7)	−0.0188 (8)
C17	0.0300 (8)	0.0353 (8)	0.0353 (9)	0.0037 (7)	−0.0130 (7)	−0.0161 (7)
C18	0.0446 (11)	0.0336 (9)	0.0457 (11)	0.0016 (8)	−0.0141 (9)	−0.0116 (8)
C19	0.0611 (14)	0.0389 (10)	0.0621 (14)	−0.0052 (10)	−0.0253 (12)	−0.0160 (10)
C20	0.0382 (10)	0.0556 (12)	0.0385 (10)	0.0054 (9)	−0.0122 (8)	−0.0258 (9)
C21	0.0411 (11)	0.0651 (14)	0.0501 (12)	0.0083 (10)	−0.0120 (9)	−0.0344 (11)

### 4,4'-(MethAnediyl)bis(3-chloro-2,6-diethylaniline) (2) Geometric parameters (Å, º)

**Table:**

Cl1—C5	1.7666 (19)	C10—H10A	0.9800
Cl2—C13	1.7614 (19)	C10—H10B	0.9800
Cl2'—C17	1.728 (6)	C10—H10C	0.9800
N1—C1	1.395 (2)	C11—C12	1.516 (3)
N1—H1A	0.8801	C11—H11A	0.9900
N1—H1B	0.8801	C11—H11B	0.9900
N2—C15	1.389 (3)	C12—C13	1.389 (2)
N2—H2A	0.8802	C12—C17	1.402 (2)
N2—H2B	0.8799	C13—C14	1.401 (3)
C1—C2	1.406 (3)	C13—H13	0.9500
C1—C6	1.410 (3)	C14—C15	1.415 (3)
C2—C3	1.389 (2)	C14—C18	1.511 (3)
C2—C7	1.513 (3)	C15—C16	1.407 (3)
C3—C4	1.401 (2)	C16—C17	1.386 (3)
C3—H3	0.9500	C16—C20	1.517 (3)
C4—C5	1.388 (2)	C17—H17	0.9500
C4—C11	1.513 (2)	C18—C19	1.529 (3)
C5—C6	1.400 (3)	C18—H18A	0.9900
C6—C9	1.506 (3)	C18—H18B	0.9900
C7—C8	1.504 (3)	C19—H19A	0.9800
C7—H7A	0.9900	C19—H19B	0.9800
C7—H7B	0.9900	C19—H19C	0.9800
C8—H8A	0.9800	C20—C21	1.531 (3)
C8—H8B	0.9800	C20—H20A	0.9900
C8—H8C	0.9800	C20—H20B	0.9900
C9—C10	1.529 (3)	C21—H21A	0.9800
C9—H9A	0.9900	C21—H21B	0.9800
C9—H9B	0.9900	C21—H21C	0.9800
			
C1—N1—H1A	113.1	C12—C11—H11A	109.3
C1—N1—H1B	111.5	C4—C11—H11B	109.3
H1A—N1—H1B	124.1	C12—C11—H11B	109.3
C15—N2—H2A	123.3	H11A—C11—H11B	107.9
C15—N2—H2B	109.2	C13—C12—C17	116.00 (17)
H2A—N2—H2B	117.7	C13—C12—C11	124.17 (16)
N1—C1—C2	119.48 (18)	C17—C12—C11	119.80 (16)
N1—C1—C6	119.95 (18)	C12—C13—C14	123.61 (17)
C2—C1—C6	120.54 (17)	C12—C13—Cl2	118.97 (14)
C3—C2—C1	118.41 (17)	C14—C13—Cl2	117.41 (14)
C3—C2—C7	122.75 (17)	C12—C13—H13	118.2
C1—C2—C7	118.81 (17)	C14—C13—H13	118.2
C2—C3—C4	123.40 (17)	C13—C14—C15	118.00 (17)
C2—C3—H3	118.3	C13—C14—C18	122.08 (18)
C4—C3—H3	118.3	C15—C14—C18	119.89 (18)
C5—C4—C3	116.10 (16)	N2—C15—C16	119.08 (18)
C5—C4—C11	124.22 (16)	N2—C15—C14	120.80 (18)
C3—C4—C11	119.66 (16)	C16—C15—C14	120.12 (18)
C4—C5—C6	123.76 (17)	C17—C16—C15	118.63 (17)
C4—C5—Cl1	118.40 (14)	C17—C16—C20	119.26 (18)
C6—C5—Cl1	117.81 (14)	C15—C16—C20	122.10 (18)
C5—C6—C1	117.77 (17)	C16—C17—C12	123.59 (17)
C5—C6—C9	121.62 (18)	C16—C17—Cl2'	107.0 (3)
C1—C6—C9	120.57 (17)	C12—C17—Cl2'	129.4 (3)
C8—C7—C2	115.95 (16)	C16—C17—H17	118.2
C8—C7—H7A	108.3	C12—C17—H17	118.2
C2—C7—H7A	108.3	C14—C18—C19	113.90 (17)
C8—C7—H7B	108.3	C14—C18—H18A	108.8
C2—C7—H7B	108.3	C19—C18—H18A	108.8
H7A—C7—H7B	107.4	C14—C18—H18B	108.8
C7—C8—H8A	109.5	C19—C18—H18B	108.8
C7—C8—H8B	109.5	H18A—C18—H18B	107.7
H8A—C8—H8B	109.5	C18—C19—H19A	109.5
C7—C8—H8C	109.5	C18—C19—H19B	109.5
H8A—C8—H8C	109.5	H19A—C19—H19B	109.5
H8B—C8—H8C	109.5	C18—C19—H19C	109.5
C6—C9—C10	111.93 (17)	H19A—C19—H19C	109.5
C6—C9—H9A	109.2	H19B—C19—H19C	109.5
C10—C9—H9A	109.2	C16—C20—C21	113.45 (16)
C6—C9—H9B	109.2	C16—C20—H20A	108.9
C10—C9—H9B	109.2	C21—C20—H20A	108.9
H9A—C9—H9B	107.9	C16—C20—H20B	108.9
C9—C10—H10A	109.5	C21—C20—H20B	108.9
C9—C10—H10B	109.5	H20A—C20—H20B	107.7
H10A—C10—H10B	109.5	C20—C21—H21A	109.5
C9—C10—H10C	109.5	C20—C21—H21B	109.5
H10A—C10—H10C	109.5	H21A—C21—H21B	109.5
H10B—C10—H10C	109.5	C20—C21—H21C	109.5
C4—C11—C12	111.77 (14)	H21A—C21—H21C	109.5
C4—C11—H11A	109.3	H21B—C21—H21C	109.5
			
N1—C1—C2—C3	176.01 (17)	C17—C12—C13—C14	−0.3 (2)
C6—C1—C2—C3	−1.9 (3)	C11—C12—C13—C14	177.54 (16)
N1—C1—C2—C7	−6.0 (3)	C17—C12—C13—Cl2	−179.42 (12)
C6—C1—C2—C7	176.03 (17)	C11—C12—C13—Cl2	−1.5 (2)
C1—C2—C3—C4	1.2 (3)	C12—C13—C14—C15	2.1 (3)
C7—C2—C3—C4	−176.68 (17)	Cl2—C13—C14—C15	−178.78 (13)
C2—C3—C4—C5	−0.1 (3)	C12—C13—C14—C18	−175.87 (16)
C2—C3—C4—C11	−178.58 (17)	Cl2—C13—C14—C18	3.2 (2)
C3—C4—C5—C6	−0.3 (3)	C13—C14—C15—N2	177.64 (16)
C11—C4—C5—C6	178.12 (17)	C18—C14—C15—N2	−4.3 (3)
C3—C4—C5—Cl1	−178.45 (13)	C13—C14—C15—C16	−2.3 (3)
C11—C4—C5—Cl1	−0.1 (2)	C18—C14—C15—C16	175.74 (16)
C4—C5—C6—C1	−0.5 (3)	N2—C15—C16—C17	−179.18 (16)
Cl1—C5—C6—C1	177.74 (14)	C14—C15—C16—C17	0.8 (3)
C4—C5—C6—C9	−178.43 (18)	N2—C15—C16—C20	0.0 (3)
Cl1—C5—C6—C9	−0.2 (2)	C14—C15—C16—C20	179.95 (16)
N1—C1—C6—C5	−176.37 (17)	C15—C16—C17—C12	1.1 (3)
C2—C1—C6—C5	1.6 (3)	C20—C16—C17—C12	−178.07 (15)
N1—C1—C6—C9	1.6 (3)	C15—C16—C17—Cl2'	−179.6 (2)
C2—C1—C6—C9	179.58 (17)	C20—C16—C17—Cl2'	1.2 (3)
C3—C2—C7—C8	6.9 (3)	C13—C12—C17—C16	−1.4 (2)
C1—C2—C7—C8	−170.95 (19)	C11—C12—C17—C16	−179.33 (16)
C5—C6—C9—C10	91.6 (2)	C13—C12—C17—Cl2'	179.5 (3)
C1—C6—C9—C10	−86.4 (2)	C11—C12—C17—Cl2'	1.6 (3)
C5—C4—C11—C12	−102.4 (2)	C13—C14—C18—C19	−96.4 (2)
C3—C4—C11—C12	76.0 (2)	C15—C14—C18—C19	85.6 (2)
C4—C11—C12—C13	−109.95 (18)	C17—C16—C20—C21	99.5 (2)
C4—C11—C12—C17	67.9 (2)	C15—C16—C20—C21	−79.7 (2)

### 4,4'-(MethAnediyl)bis(3-chloro-2,6-diethylaniline) (2) Hydrogen-bond geometry (Å, º)

*Cg*1 and *Cg*2 are the centroids of the C1–C6 and C12–C17 rings, respectively.

**Table:**

*D*—H···*A*	*D*—H	H···*A*	*D*···*A*	*D*—H···*A*
N2—H2*A*···*Cg*1^i^	0.88	2.63	3.327 (2)	137
C7—H7*B*···*Cg*2^ii^	0.99	2.83	3.579 (2)	133
C20—H20*B*···*Cg*2^i^	0.99	2.81	3.434 (2)	132

Symmetry codes: (i) −*x*+1, −*y*+1, −*z*+1; (ii) *x*+1, *y*, *z*.
